# C subunit of the ATP synthase is an amyloidogenic calcium dependent channel-forming peptide with possible implications in mitochondrial permeability transition

**DOI:** 10.1038/s41598-021-88157-z

**Published:** 2021-04-22

**Authors:** Giuseppe Federico Amodeo, Brenda Yasie Lee, Natalya Krilyuk, Carina Teresa Filice, Denis Valyuk, Daniel Erik Otzen, Sergey Noskov, Zoya Leonenko, Evgeny V. Pavlov

**Affiliations:** 1 Department of Molecular Pathobiology, New York University College of Dentistry, 345 East 24th Street, New York, NY 10010-4086 USA; 2 Department of Physics and Astronomy, University of Waterloo, Waterloo, Canada; 3 Department of Biology, University of Waterloo, Waterloo, Canada; 4 Interdisciplinary Nanoscience Center (iNANO) and Department of Molecular Biology and Genetics, Aarhus University, Aarhus C, Denmark; 5 Centre for Molecular Simulation and Biochemistry Research Cluster, Department of Biological Sciences, University of Calgary, Calgary, Canada

**Keywords:** Biochemistry, Biophysical chemistry, Protein folding, Proteins, Structural biology, Biophysics, Bioenergetics, Membrane structure and assembly, Molecular biophysics, Permeation and transport, Single-molecule biophysics, Chemical biology, Biophysical chemistry, Ion channels, Protein folding, Proteins, Structural biology, Atomic force microscopy

## Abstract

The c subunit is an inner mitochondrial membrane (IMM) protein encoded by three nuclear genes. Best known as an integral part of the F_0_ complex of the ATP synthase, the c subunit is also present in other cytoplasmic compartments in ceroid lipofuscinoses. Under physiological conditions, this 75 residue-long peptide folds into an α-helical hairpin and forms oligomers spanning the lipid bilayer. In addition to its physiological role, the c subunit has been proposed as a key participant in stress-induced IMM permeabilization by the mechanism of calcium-induced permeability transition. However, the molecular mechanism of the c subunit participation in IMM permeabilization is not completely understood. Here we used fluorescence spectroscopy, atomic force microscopy and black lipid membrane methods to gain insights into the structural and functional properties of unmodified c subunit protein that might make it relevant to mitochondrial toxicity. We discovered that c subunit is an amyloidogenic peptide that can spontaneously fold into β-sheets and self-assemble into fibrils and oligomers in a Ca^2+^-dependent manner. C subunit oligomers exhibited ion channel activity in lipid membranes. We propose that the toxic effects of c subunit might be linked to its amyloidogenic properties and are driven by mechanisms similar to those of neurodegenerative polypeptides such as Aβ and α-synuclein.

## Introduction

The c subunit is a short (75 residues) peptide whose sequence is highly conserved from bacteria to humans. In eukaryotes, this highly hydrophobic peptide is a transmembrane α-helical “hairpin” localized in the mitochondrial inner membrane where it assembles into oligomers of 8–16 units depending on the species. Under normal physiological conditions oligomers of the c subunit are an integral part of the F_0_ complex of the ATP synthase where they form the c-ring that is the main constituent of the rotor^1^. The c subunit can also be found in cytosolic compartments and the plasma membrane where it accumulates in excessive amounts in ceroid-lipofuscinoses (lysosomal storage or Batten disease)^2,3^.

It has recently been proposed by several groups that, in addition to its normal physiological function, the c subunit may play a critical role in mitochondrial pathology by participating in calcium-induced permeability transition (PT)^4–6^. PT is a phenomenon of increased inner mitochondrial membrane (IMM) permeability by the mechanism of opening of the PT pore in response to toxic levels of reactive oxygen species and/or calcium^7–9^. PT is believed to be a major contributor to cell and tissue damage under conditions of acute stress, including ischemia–reperfusion injury. While multiple experimental studies support the involvement of the c subunit in PT, the molecular basis of its participation remains poorly understood and is a subject of considerable debate^10–14^. Indeed, in its physiological “c-ring” assembly c subunit is not expected to allow ion flux due to the highly hydrophobic environment within its lumen^15^. However, fractions containing c subunit extracted from mitochondria exhibit channel activity in model lipid bilayers^4,6,16^ suggesting the possibility of its direct participation in PT through the formation of an ion conducting pore in pathologies. We hypothesized that ion conducting capability of c subunit might involve conformational rearrangements and/or assemblies present not in basal but rather in pathological conditions. Here we investigated this possibility by testing whether the unmodified mature c subunit protein could form channels in model lipid bilayers. To exclude potential contributions of other biological compounds, a synthetic c subunit protein was used. We combined a structural and functional approach to characterize the peptide and test its channel forming properties. Our study found that the c subunit is an amyloidogenic peptide that has the ability to fold into a β-sheet conformation and form fibrils or oligomers in a calcium dependent manner. These observed oligomers can form stable pores in model lipid bilayers. We propose the possibility that in addition to its role in native conditions, c subunit could exert a pathological action on mitochondria through its misfolded oligomers. Based on these findings, we hypothesize that the mechanism of c subunit toxicity on mitochondrial membranes occurs through structural rearrangements similar to those described for other amyloidogenic polypeptides including Aβ (Alzheimer’s Disease) and α-synuclein (Parkinson’s Disease) and possibly other synucleopathies^17–19^.

## Materials and methods

### Chemicals

Synthetic peptides with free termini were purchased from LifeTein Inc. (Somerset, NJ, USA) with a purity of 98%. The lyophilized peptides were dissolved in increasing volumes of 2% Genapol and phosphate buffer saline until the saturation point (50 mM) was reached. The working concentrations for the experiments were then adjusted to this concentration.

### Circular dichroism

CD spectra of synthetic c subunit were recorded on a Jasco J-1500 spectropolarimeter (Jasco Corporation, Japan) with a 1 mm path length cell. Spectra were recorded in the spectral range of 180 − 260 nm, with a scan rate of 10 nm/min at 0.5 nm intervals. Data were acquired at 25 °C, and 10 scans were averaged for each spectrum. Peptide CD spectra were collected in 2% Genapol, which is a non-ionic and non-polar detergent best suited for these experiments and phosphate buffer saline pH 7 at a final concentration of 30 µM.

### BLM recordings

The painting method was used to form phospholipid bilayer using 1,2-diphytanoyl-sn-glycerol-3-phosphocholine (DiPhPC, Avanti Polar Lipids). Bilayer was formed at the 50–100 µm diameters apertures of Delrin cuvettes as previously described^20^. Briefly, the aperture was pre-treated with 25 mg/mL of DiPhPC in decane and allowed to dry. Bilayers were formed using the painting method after filling up the cuvettes with the recording solution (150 mM KCl, 20 mM HEPES, pH 7.4) on both sides of the chamber. Ion currents were measured using standard Ag-AgCl electrodes from WPI (World Precision Instruments) that were placed in each side of the cuvette. Measurements of the conductance of single channels were performed by painting the protein to the cis side of the chamber (the side connected to the ground electrode). Spontaneous channel insertion was typically obtained under an applied voltage of 20 mV. Conductance measurements were performed using an eONE amplifier (Elements) with a sampling rate of 10 kHz (809.1 µs interval). Traces were filtered by low-pass Bessel filter at 10 Hz for analyses performed with Origin Pro 2021 (OriginLab Corp.) and Clampfit software (Molecular devices).

### Thioflavin T assay

Fresh stock solutions of c subunit in 2% Genapol/PBS were prepared at room temperature and transferred into a clear-bottomed 96-well plate (CELLSTAR, GBO, Austria) at a final concentration of 5 µM together with 30 µM thioflavin T. Readings were conducted in triplicates either with or without 1 mM Ca^2+^. The plate was loaded into a Flexstation 3 microplate reader (Molecular Devices, San Jose, CA) and incubated at 37 °C without agitation for 20 h. The fluorescence was measured at 30-s intervals, with excitation at 440 nm and with emission at 480 nm.

### AFM imaging

The JPK/Bruker Nanowizard II atomic force microscope was used to image aggregates of c subunit deposited on mica slides. To form aggregates samples were pre-incubated for 4 h at 37 °C with or without 1 mM CaCl_2_ under conditions identical to the ThT assay. Deposited aggregates samples were prepared for imaging as following: small aliquots (10 mL, 15 mL and 20 mL) of the aggregate solution were deposited onto the freshly cleaved surface of the mica slide, incubated for 10 min, rinsed with ultrapure water and dried with a gentle stream of nitrogen prior to imaging. NCH AFM cantilevers were purchased from NanoWorld (Neuchâtel, Switzerland), designed for non-contact and tapping mode imaging to offer high sensitivity and speed while scanning (320 kHz resonance frequency, 42 N/m force constant, thickness 4 mm, no coating). AFM imaging was done in air in intermittent contact mode. A minimum of three samples made were used for statistical analysis, with at least six 5 × 5 µm images obtained for each sample at a resolution of 2048 pixels, and at least 100 measurements were used for statistical analysis of height and diameters calculated.

### SDS-PAGE

Gradient 4–20% Tris-TGX gels were used. Prior to loading, the samples were incubated for 2 h at room temperature with and without calcium. Next, samples were treated with 5 volumes of 12% (wt/vol) trichloroacetic acid solution and incubated on ice for 5 min. The pellet was collected by centrifugation (11,000×*g* for 5 min at 4 °C) and dissolved in 2% (wt/vol) SDS-containing loading buffer. Prior to being loaded on an SDS-gel, samples were neutralized by the addition of 1 µL aliquots of 0.5 M NaOH until the color of the bromophenol blue-stained loading buffer turned from yellow to blue and were then heated at 98 °C for 10 min^21^. The separation was performed by applying a constant current of 30 mA. Finally, the gels were stained with silver.

### Dot blot

Freshly prepared synthetic c subunit aliquots were incubated with or without 1 mM Ca^2+^ at 37 °C for 4 h and 0.66 μg of samples were spotted on a polyvinylidene difluoride (PVDF) membrane. Proteins were blocked in 0.15 M TrisHCl, 0.45 M NaCl, 1% Triton (TBS-A) and 5% nonfat dry milk for 1 h at RT. Membranes were then incubated with c subunit (ab180149) and A11-19 antibodies at 1:2000 and 1:1000 dilutions respectively. Following three washes with TBS-A, membranes were incubated in goat anti-rabbit anti-IgG (H + L)-HRP conjugated at 1:3000 dilution for 1 h at RT. Bands were detected with the Pierce ECL Western Blot Substrates and visualized with the Biorad Gel Doc EQ system.

### Bioinformatic analyses

The secondary structure predictions were performed using the Chou and Fasman secondary structure prediction server (CFSSPS) applying a window of three residues^22,23^.

## Results

### C subunit spontaneously folds into a β-sheet conformation

**Figure 1:**
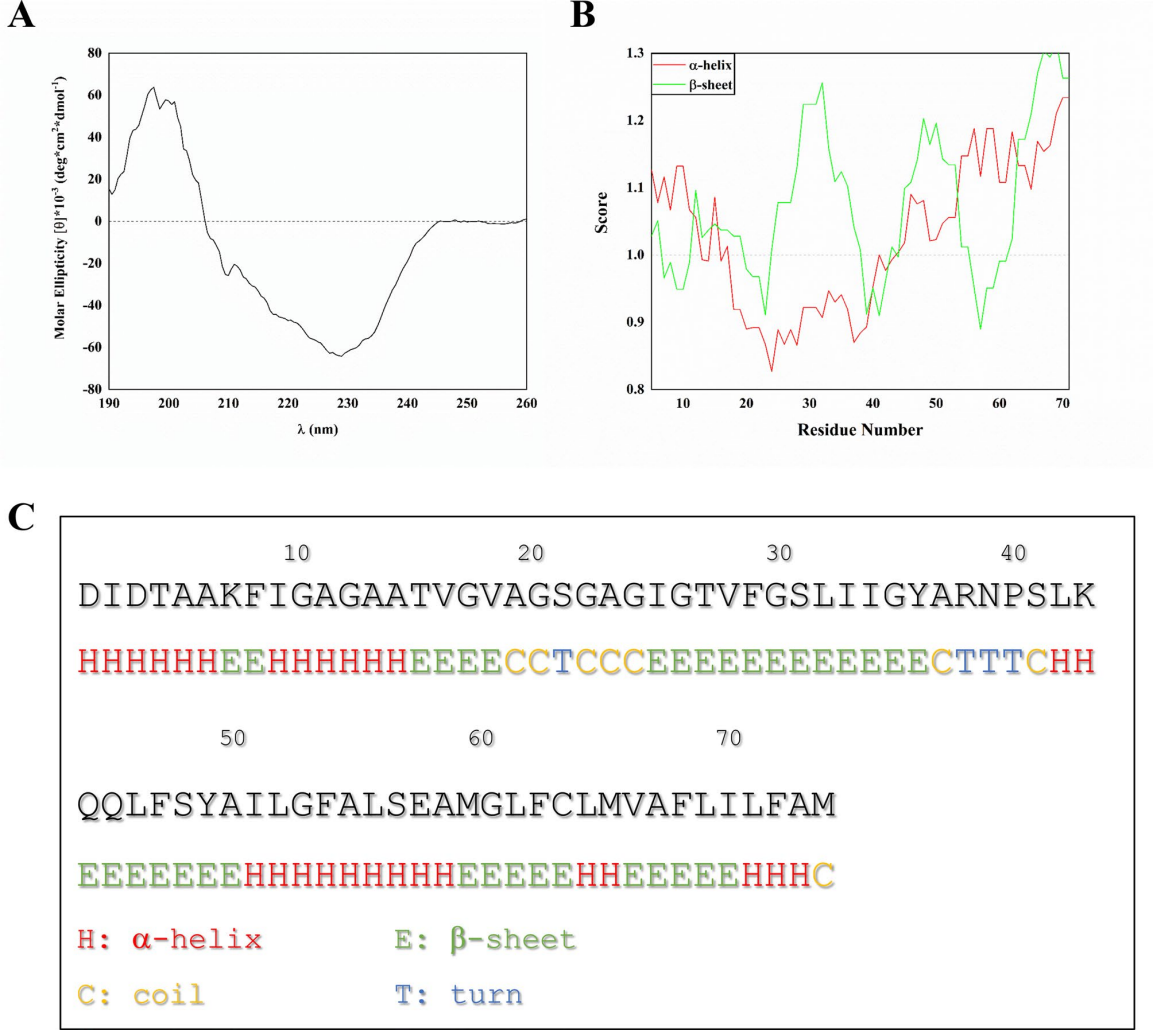
C subunit forms β-sheet structures. (**A**) CD spectrum of the 50 µM c subunit in 2% Genapol and PBS; (**B**) Bioinformatic prediction of the probability score of the secondary structure of the c subunit based on its primary sequence according to the Chou and Fasman method; (**C**) Most probable predicted structure per individual residue (Created with OriginPro 2021, OriginLab Corp. Northampton, MA).

To investigate the conformational properties of c subunit, we dissolved lyophilized synthetic peptide in a buffer containing a high concentration of a non-ionic surfactant to mimic a membrane-like environment (2% Genapol and phosphate buffer saline (PBS) at pH 7). Genapol is a non-ionic detergent and is much less disruptive of native peptide/protein structure than ionic detergents such as SDS, and in fact tend only to interact with proteins and peptides which are normally embedded in membranes. In practice this makes them much better membrane mimics than SDS^24^. After incubation for 15–24 h, we recorded the peptide’s far-UV circular dichroism (CD) spectrum (Fig. 1A). The CD spectrum differs markedly from the clear α-helical features of the c subunit found in native conditions, i.e. minima around 209 and 222 nm and a maximum around 193 nm. Instead, there is a maximum around 198 nm and a minimum around 226 nm. These values are higher than a canonical β-sheet, which typically shows a minimum around 215 nm and a maximum around 195 nm. This suggests additional contributions from other conformations. A CD-spectrum enabled secondary structure analysis performed with BestSel^25^ software suggested that membrane-inserted c subunit oligomers are comprised of ≈ 44% of antiparallel β-strands and the remaining ≈ 56% being either turns or other conformations such as β-bridges, bends or unordered structures. Our results are consistent with a secondary structure prediction based solely on the primary sequence of the c subunit and unbiased by existing structures (Fig. 1B,C). The prediction indicates that while the probability of folding into a β-sheet is uniformly distributed along the sequence, there is a gap between A14 and A50 where the score for the α-helix is very low. These results suggest the possibility that similar to some other amyloidogenic polypeptides, if the c subunit is not folded into its native conformation by a functional protein folding machinery it might enter a misfolding pathway that leads to β-sheets.

### Fibrillation and Ca^2+^-induced oligomerization of the c subunit

**Figure 2:**
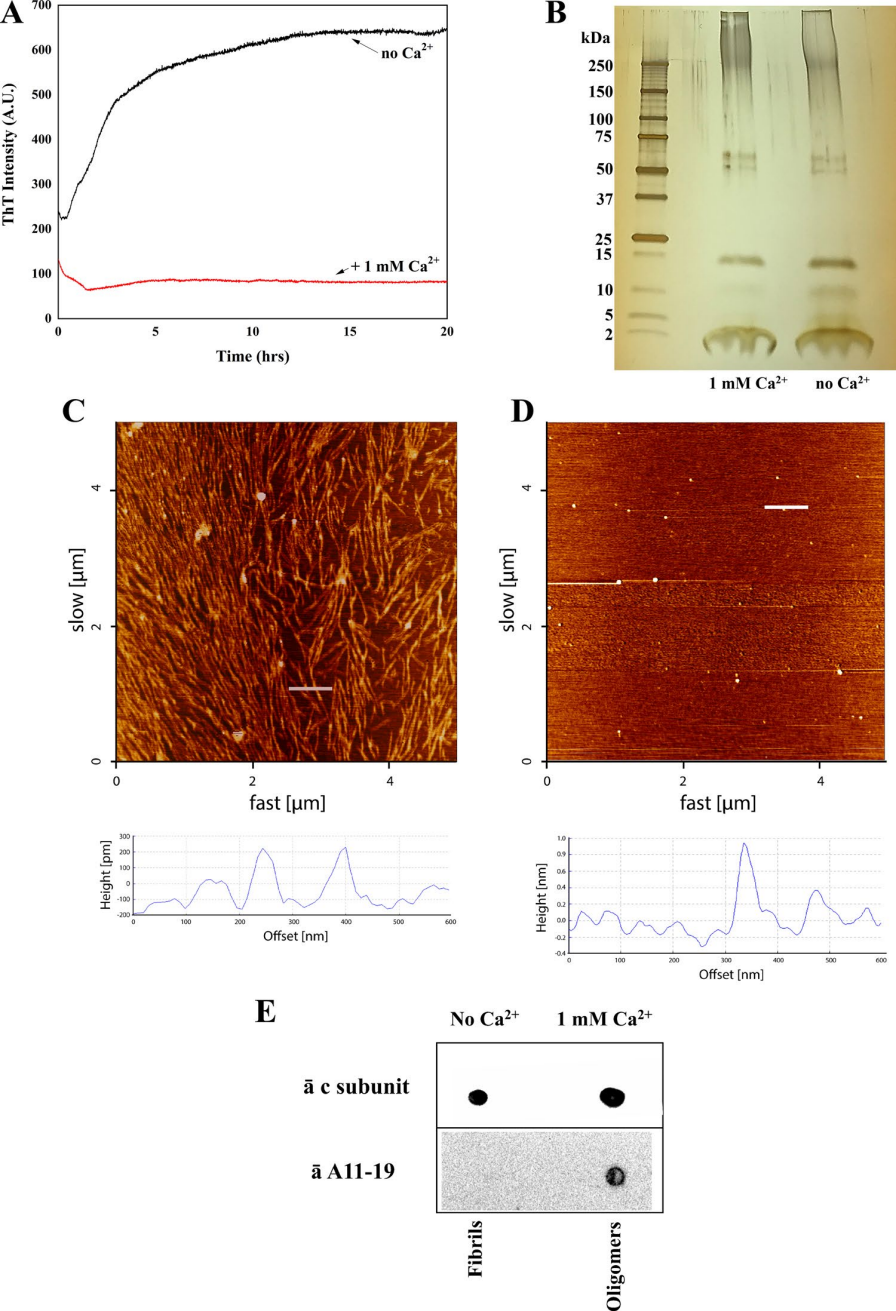
Aggregation properties of the c subunit. (**A**) Thioflavin T fluorescence spectra showing calcium dependence of c subunit aggregation (created with OriginPro 2021, OriginLab Corp. Northampton, MA); (**B**) SDS PAGE of the c subunit showing presence of oligomeric and monomeric forms; (**C**) 5 × 5 µm AFM image of c subunit fibrils after incubation at 37 °C for 4 h in 2% Genapol and PBS; (**D**) 5 × 5 µm AFM image of the c subunit oligomers (arrows) after incubation at 37 °C for 4 h and deposited on mica. Below each image is provided the height profile of the indicated white line; (**E**) Dot blot of the c subunit incubated with anti c subunit and A11-19 antibodies with and without Ca^2+^ showing the ability of the protein to form cross-β oligomers (full-length blots are presented in Supplementary Figure 1).

Next we tested if, similarly to other amyloidogenic peptides, the c subunit in β-sheet conformation could form aggregates. We therefore monitored the formation of cross-β aggregates using the amyloid-binding dye Thioflavin T (ThT). In the absence of Ca^2+^ ions, there was a rapid rise in ThT fluorescence within 3–4 h at 37 °C, which reached a plateau level around 10–15 h after the start (Fig. 2A). The aggregation process was strongly suppressed by 1 mM Ca^2+^ (Fig. 2A). The tendency of the c subunit to form complexes was further confirmed by gel electrophoresis (Fig. 2B) which shows that the c subunit preparation contains a number of oligomers with MW ranging from 15 up to 250 kDa. Importantly these assemblies were present in both Ca^2+^ and Ca^2+^-free samples, suggesting that calcium ion inhibits fibril formation rather than inducing oligomerization. To visualize the ultrastructure of these c subunit assemblies, we performed atomic force microscopy (AFM) imaging of c subunit samples pre-incubated for 4 h at 37 °C under conditions identical to the ThT assay and deposited on mica. In agreement with CD and ThT data, AFM images of the c subunit samples prepared in the absence of Ca^2+^ showed densely packed fibril structures. The typical diameter estimated from the height measurements of these fibrils was around 20 nm (22.2 ± 0.9 nm) and the length spanning in micrometer range (Fig. 2C). In contrast, the presence of 1 mM Ca^2+^ led to the formation of small spherical oligomers with diameters around 60 nm (67.6 ± 1.4 nm) and few larger aggregates and, importantly, completely inhibited formation of fibrils (Fig. 2D). Based on the bands observed in the electrophoretic run and the thickness of the membrane from AFM data, we estimate that at least 8 c subunits would be necessary to assemble into cross-b oligomers. Further, the dot blot of the samples showed a signal for cross-β amyloid-like oligomers in the presence of Ca^2+^ (Fig. 2E). These assays demonstrate a critical role for Ca^2+^ in the folding and self-assembly of c subunits into oligomers rather than fibrils and support the possibility that a similar mechanism may be associated to the c subunit.

### Ion channel activity of c subunit oligomers

**Figure 3:**
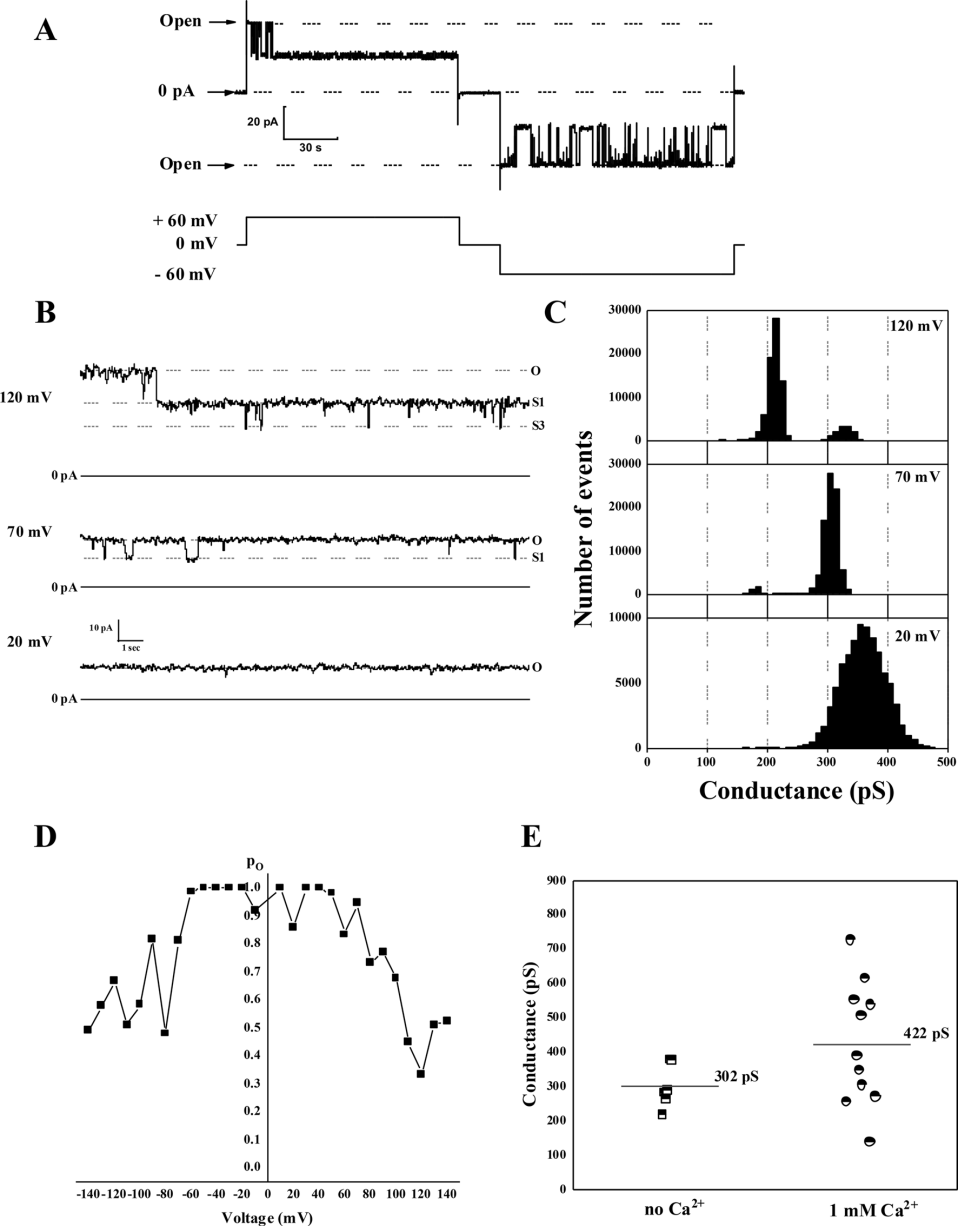
Ion channel activity of c subunit oligomers. (**A**) Representative current trace of oligomers showing typical channel behaviour with frequent transitions between fully open and lower conductance states; (**B**) Representative c subunit channel activity at different voltages; (**C**) All points histogram corresponding to the trace shown at panel (**B**); (**D**) Voltage dependence of the open probability of the c subunit channel; (**E**) Channel open state conductance values of the c subunit channels from multiple independent experiments alone (n = 5) and in the presence of Ca^2+^ (n = 11) (Created with OriginPro 2021, OriginLab Corp. Northampton, MA).

One of the most critical properties that underlie the toxicity of amyloidogenic peptides is their ability to form ion channels in lipid bilayers^26^. Considering the implications of c subunit in mitochondrial membrane permeabilization and after establishing its amyloidogenic properties we tested its channel forming activity. To do this we reconstituted the preparations described in the previous section into artificial planar lipid bilayers and measured their electrical currents enabled by voltage clamping. We observed that both samples prepared with and without Ca^2+^ exhibit robust ion currents. Figure 3A shows representative ion channel behavior of the c subunit observed in our experiments. These channels show multiple conductance states and were voltage dependent with a tendency to switch to the low conductance states at higher voltages (Fig. 3B–D). The channel activity was similar in both preparation with slight cation selectivity (P_K_/P_Cl_ = 6 ± 2, n = 8) and an average conductance ranging from 300 to 400 pS. The point distributions of the channel conductances (Fig. 3E) show slightly lower values for the channels from the preparations containing fibrils, but the difference was not statistically significant (p = 0.13). The similarity between channel activities of preparations with or without Ca^2+^ suggests that pore forming assembly is not related to the fibrils but rather to oligomers which are present in both samples (Fig. 2B). This behavior is consistent with other known channel-forming amyloidogenic peptides^26,27^.

## Discussion

Here we report that the human unmodified synthetic c subunit is an amyloidogenic peptide and its oligomers are capable of forming ion conducting pores in planar lipid bilayers.

Due to the tendency of the peptide not to stay as monomers but to self-aggregate it was not possible to test specifically the effects of monomers on bilayer conductance. However, taking into account the relatively small size of the peptide and its high hydrophobicity we propose that it is very unlikely that monomers will be able to cause pore-like ion conductance.

PT occurs upon formation of the PTP that may require the participation of various protein complexes including the c subunit^4,28^, ATP synthase dimers^29^ and the adenine nucleotide transferase (ANT)^30^. It is currently not entirely clear which conditions favor one type of PTP or the other. At this stage we can only speculate that the molecular pathway of PT would depend on the mechanism of induction and/or cell type.

One of the possible mechanisms behind the participation of the c subunit in PTP formation may be associated with the Aβ and α-synuclein pathways^31^. These peptides have been found in mitochondria with high levels of Ca^2+^ and ROS. Similar to our findings, Aβ and α-synuclein alone in their oligomeric conformations are able to form ion channels in planar lipid bilayers with lower conductances compared to the canonical PTP. An early study on the purified c subunit from brain or plants membranes showed indeed the ability of this protein to undergo into conformational transitions leading to β-sheet formation^32^.

It has to be noted that the formation of the PTP due to the import of amyloidogenic peptide does not exclude the ability of mitochondrial endogenous membrane proteins to participate in PT by permeabilizing the IMM. In fact, our data and those for other amyloidogenic proteins suggest the existence of a distinct pathway that may be likely disease dependent^31^. Specifically, series of experimental data have shown that these peptides are imported into the mitochondrial matrix where they oligomerize and induce the permeabilization of the IMM^31^. Altogether, these data raise the possibility that c subunit can form channels, while not being part of ATP synthase complex, with its monomers misfolding and assembling into the toxic oligomers in a process regulated by chaperones and lipids^33^. It has been previously reported that c subunit alone without other parts of ATP synthase is sufficient to induce PT^5,34^. Further, c subunit extracted from mitochondria can form channels in lipid bilayers^6,35^. However, physicochemical properties of c subunit in its native conformation when this peptide is highly hydrophobic are not consistent with an ionophoretic function which would require presence of the hydrophilic water-filled pore region^15^. It has been argued that the channel forming activity might require the presence of other components that co-purify with the c subunit or post-translational modifications. Our findings indicate that the c subunit alone is sufficient to form ion channels but in order to do so it needs to assemble into oligomers in a β-sheet conformation. We hypothesize that the overall mechanism of the c subunit permeabilization of the inner mitochondrial membrane might be similar to the mechanism of some other amyloidogenic peptides that form β-sheet oligomeric pores^36,37^. Interestingly, a recent study estimated that during permeability transition a very few mPTP channels are activated in each mitochondrion^38^. Thus, if the β-sheet pathway is activated a small fraction of these misfolded peptides will be sufficient to induce permeability transition. On a special note, the possibility of monomers in α-helix conformation permeabilizing membranes has been theorized however this is unlikely the case for the c subunit due to its high hydrophobicity in such conformation^19,39–41^. Our work was focused specifically on the synthetic peptide that allowed us to exclude contributions of other biological molecules while focusing on the unmodified c subunit. We also used synthetic lipid bilayer to exclude possible effects of the lipid composition of the membrane, which is known to modulate toxicity of other amyloids and will be important to investigate in the future^40^. Despite this we don’t exclude that such contributions may play a relevant role in the toxicity of the c subunit; in fact, there is a significant possibility that similar conformational changes might occur in vivo. Specifically, it has been established that, similar to other amyloidogenic peptides, c subunit tends to form aggregates both in vivo and in vitro supporting the notion that it converts into a toxic form under pathological conditions^2,32,35^. Importantly, in our experiments we studied the unmodified c subunit peptide while in vivo most of the c subunit is found in methylated form in lysine-43 position^42^. The c subunit is encoded by three nuclear genes, namely ATP5G1, ATP5G2 and ATP5G3, that express the same mature protein with differing signal peptides for each. After expression the unfolded protein is imported into the mitochondrial matrix, the signal peptide is cleaved and the mature sequence gets processed for folding. A recent study identified the methylase responsible for this post-translational modification (PTM) in the mitochondrial FAM173B showing that in cells lacking this enzyme, the c subunit could still form protein complexes but would not incorporate into the ATPase resulting in a reduction of respiration and ATP production^43^. However, there may be a small pool of unfolded c subunit (i.e. freshly imported protein) that due to the high levels of Ca^2+^ and the lack of a functional folding machinery spontaneously folds into a β-sheet conformation. Based on these data, it is very likely that this PTM directly induces folding of the c subunit into the native α-helical hairpin whilst its absence leads to protein aggregation. Most importantly, this study supports the hypothesis that the unfolded c subunit is responsible for ion channel activity and is in agreement with the notion that only few pores are open during PT.

Interestingly, we found that fibril formation was dependent on the presence of Ca^2+^. At this point the relationship, if any, between this phenomenon and PT is not clear especially taking into account that oligomers were detected regardless of the presence of Ca^2+^.

**Figure 4:**
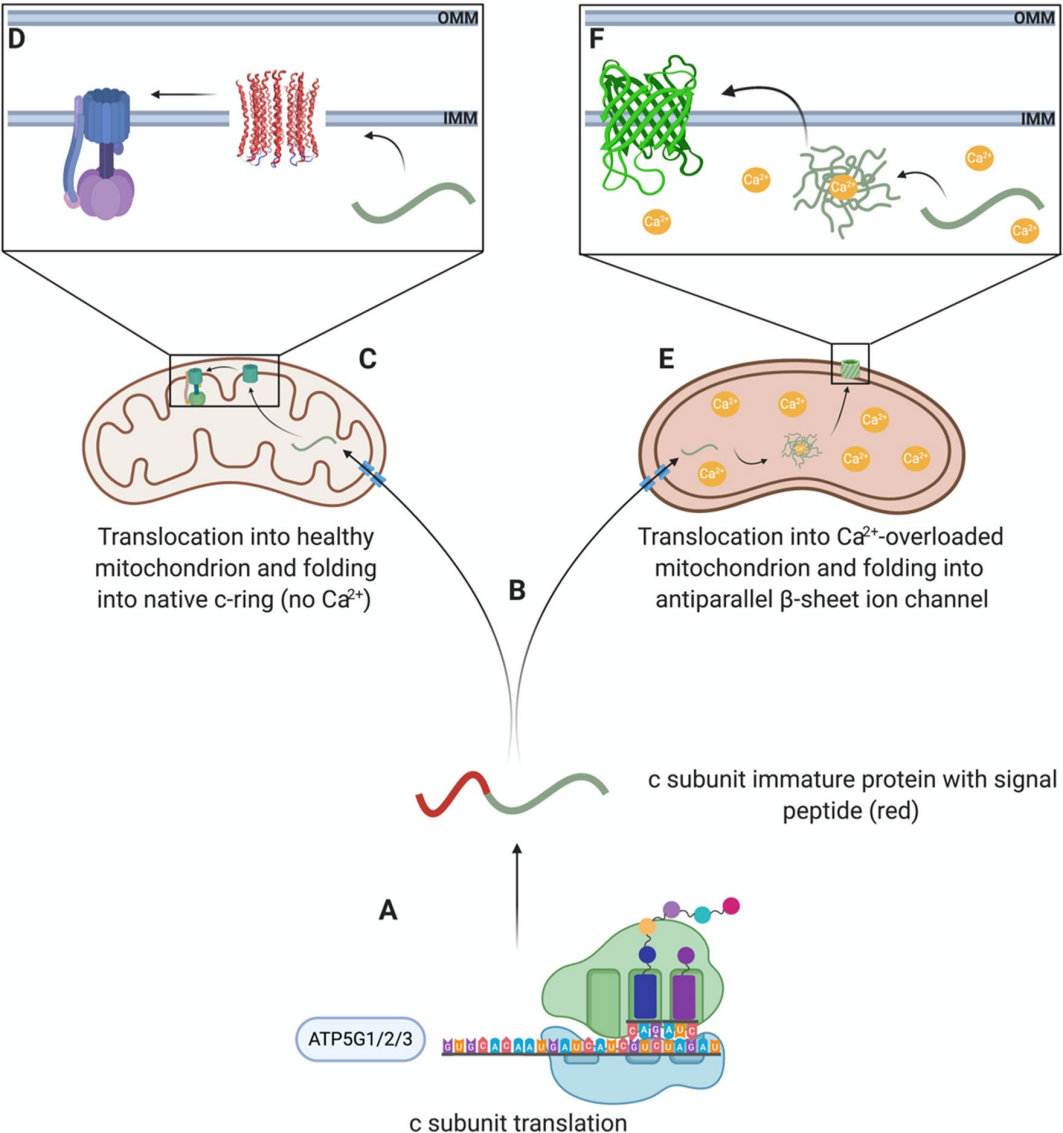
Function of c subunit in physiological and pathological mitochondria. The c subunit is encoded by three nuclear genes: ATP5G1, ATP5G2 and ATP5G3. Each of these genes, when translated, express the same mature sequence of the c subunit with differing signal peptides (**A**). Predictions based on the immature sequences of the c subunit isoforms indicate that the protein may be imported into mitochondria through the TIM/TOM complex (**B**). In physiological conditions (**C**), c subunit monomers fold into their native α-helical hairpin conformation and oligomerize into the c-ring of the ATPase. Conversely, in pathological conditions (**E**), the increased levels of Ca^2+^ influence the misfolding of the newly imported peptides causing them to oligomerize into a cross-β conformation (**F**) that contributes to PTP through permeabilizing the IMM (Created with Biorender.com).

In summary we report for the first time that the c subunit in its unmodified form is an amyloidogenic peptide capable of self-assembly into β-sheet oligomers and that its observed ionophoretic properties are linked to its amyloidogenic nature. We propose that these toxic forms of misfolded c subunit might play a significant role in cell pathophysiology (Fig. 4).

## Supplementary Information

Supplementary Figure 1.

## Data Availability

The data that support the findings of this study are available on request.
