# Supplementary material for: C subunit of the ATP synthase is an amyloidogenic calcium dependent channel-forming peptide with possible implications in mitochondrial permeability transition
Source: Sci Rep. 2021 Apr 22;11:8744. doi: 10.1038/s41598-021-88157-z (PMC8062469; doi:10.1038/s41598-021-88157-z)
Supplement: Supplementary file 1 — Supplementary Figure 1. [file 41598_2021_88157_MOESM1_ESM.pdf]

C subunit of the ATP synthase is an amyloidogenic calcium dependent channel-forming peptide with possible implications in mitochondrial permeability transition

Giuseppe Federico Amodeo[1], Brenda Yasie Lee[2], Natalya Krilyuk[1], Carina Teresa Filice[2,3], Denis Valyuk[1], Daniel Erik Otzen[4], Sergey Noskov[5], Zoya Leonenko[2,3], Evgeny V. Pavlov[1,*]

[1]Department of Molecular Pathobiology, New York University, [2]Department of Physics & Astronomy, University of Waterloo, Canada, [3]Department of Biology, University of Waterloo, Canada, [4]Interdisciplinary Nanoscience Center (iNANO) and Department of Molecular Biology and Genetics, Aarhus University, Aarhus C, Denmark, [5]Centre for Molecular Simulation and Biochemistry Research Cluster, Department of Biological Sciences, University of Calgary, Canada

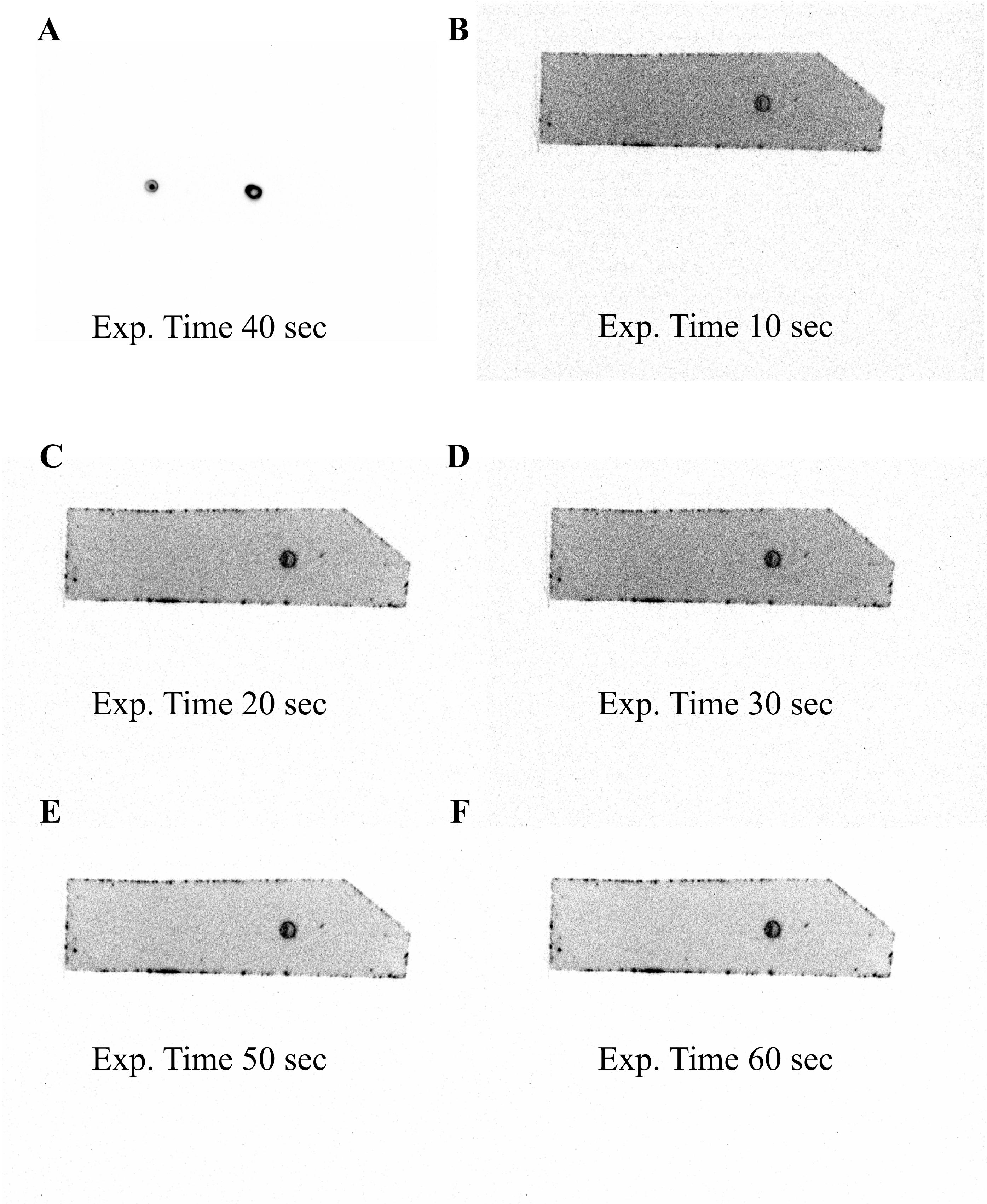

Supplementary Figure 1: Dot blots of the unmodified c subunit incubated in absence and in presence of Ca^{2+} using (A) anti c subunit antibodies and (B-F) A11-19 antibodies at different exposures (reported below every blot).
